# Supplementary material for: Emergence and antigenic characterisation of influenza A(H3N2) viruses with hemagglutinin substitutions N158K and K189R during the 2024/25 influenza season
Source: bioRxiv. 2026 Feb 10:2026.02.10.704996. Preprint. [Version 1] doi: 10.64898/2026.02.10.704996 (PMC12918802; doi:10.64898/2026.02.10.704996)
Supplement: Supplement 1 [file media-1.pdf]

Supplementary material

This supplementary material is hosted by Eurosurveillance as supporting information alongside the article [Title], on behalf of the authors, who remain responsible for the accuracy and appropriateness of the content. The same standards for ethics, copyright, attributions and permissions as for the article apply. Supplements are not edited by Eurosurveillance and the journal is not responsible for the maintenance of any links or email addresses provided therein.

Supplemental Table 1

Data Availability

GISAID Identifier: EPI_SET_250402wk

DOI: https://doi.org/10.55876/gis8.250402wk
All genome sequences and associated metadata in this dataset are published in GISAID's EpiFlu database. To view the contributors of each individual sequence with details such as accession number, Virus name, Collection date, Originating Lab and Submitting Lab and the list of Authors, visit 10.55876/gis8.250107tk

Data Snapshot
EPI_SET 250402wk is composed of 27111 individual viruses.
The collection dates range from 2013-12-06 to 2025-03-18;
Data were collected in 145 countries and territories.

Supplemental Table 2

Data Availability

GISAID Identifier: EPI_SET_251001mv

DOI: https://doi.org/10.55876/gis8.251001mv
All genome sequences and associated metadata in this dataset are published in GISAID's EpiFlu database. To view the contributors of each individual sequence with details such as accession number, Virus name, Collection date, Originating Lab and Submitting Lab and the list of Authors, visit EPI_SET_251001mv

Data Snapshot
EPI_SET_251001mv is composed of 594 individual viruses.
The collection dates range from 2024-09-30 to 2025-03-09;
Data were collected in 1 countries and territories.

Supplemental Table 3. Hemagglutination inhibition assay results of A(H3N2) virus variants using glycan-modified erythrocytes.

	Characterizing amino acid substitution(s)	A/NL/00322/2020	A/NL/00652/2023	A/TH/8/2022	A/DC/27/2023	A/CR/10136RV/2023	A/NL/01285/2024	A/NO/423/2024	A/SL/49/2024
A/NL/00322/2020		**1280**	80	30	160	20	<10	<10	160
A/NL/00652/2023		320	**1280**	1280	1920	1280	640	640	480
A/TH/8/2022		1280	2560	**3840**	5120	3840	1280	1280	1280
A/DC/27/2023	S145N	80	320	480	**1280**	1280	320	320	320
A/CR/10136RV/2023	S145N	160	320	320	640	**640**	320	320	320
A/NL/01285/2024	K189R	10	160	160	640	320	**640**	20	80
A/NO/423/2024	N158K	640	640	2560	1280	640	640	**15360**	20480
A/SL/49/2024	N158K	160	320	640	640	240	480	10240	**20480**
A/NL/10038/2025	S145N	80	240	320	640	320	320	320	240
A/NL/10067/2025	S145N	40	320	320	640	960	240	320	320
A/NL/10737/2024	T135K + S145N	40	320	640	960	640	160	160	480
A/NL/01809/2024	T135K	240	960	1280	2560	2560	480	640	1280
A/NL/01829/2024	K189R	10	120	240	640	320	320	<10	40
A/NL/01577/2024	N158K	120	320	640	640	160	320	5120	10240
A/NL/00520/2025	N158K + K189R	20	80	40	80	40	320	1280	1280
A/NL/02093/2024	N158K + K189R	20	80	40	80	40	320	1280	1280
A/NL/00024/2025	N158K + K189R	30	160	40	80	80	640	1280	1280
A/NL/00328/2025	N158K + K189R	20	120	40	80	60	480	1280	1280
A/NL/10685/2024	N158K + K189R	*	*	20	*	*	160	640	640
A/NL/10103/2025	N158K + K189R	10	80	20	40	40	320	1280	1280

CR, Croatia; DC, District of Columbia; NL, Netherlands; NO, Norway; SL, Slovenia; TH, Thailand.

The antisera (columns) are designated according to the virus (rows) used to prepare the antiserum. An asterisk indicates that the virus – antiserum combination was not tested. When the highest concentration of antiserum did not inhibit hemagglutination, the titer is indicated as lower than the lowest dilution tested (e.g. <10). Homologous titers are shown in bold.

Supplemental Table 4. Hemagglutination inhibition assay results of A(H3N2) virus variants using guinea pig erythrocytes.

	Characterizing amino acid substitution(s)	A/TH/08/2022	A/DC/27/2023	A/CR/10136RV/2023	A/NL/10563/2023	A/LI/216/2023	A/SL/49/2024	A/SW/47775/2024	A/NL/10685/2024	A/CA/NSVH102423723/2024
A/MA/18/2022		320	320	320	320	320	160	40	<40	160
A/TH/08/2022		**320**	640	640	320	640	320	160	40	320
A/DC/27/2023	S145N	160	**320**	640	320	320	320	160	40	160
A/CR/10136RV/2023	S145N	320	640	**1280**	640	640	640	160	80	640
A/NL/10563/2023	S124N	160	320	640	**640**	320	320	160	40	320
A/LI/216/2023	S124N	320	640	640	320	**320**	160	80	<40	320
A/SL/49/2024	N158K	40	80	160	80	160	**1280**	40	640	80
A/SW/47775/2024	K189R	<40	160	80	80	40	<40	**160**	<40	80
A/NL/10685/2024	N158K + K189R	<40	<40	<40	<40	80	320	40	**640**	<40
A/CA/NSVH/2024	T135K	320	320	320	320	320	320	80	40	**1280**
A/NL/00520/2025	N158K + K189R	<40	<40	<40	<40	80	640	40	1280	40
A/NL/00401/2025	N158K + K189R	<40	40	40	<40	80	640	40	1280	40
A/NL/00328/2025	N158K + K189R	<40	40	80	40	80	640	40	1280	80
A/NL/00077/2025	N158K + K189R	<40	<40	<40	<40	80	640	40	640	40
A/NL/00156/2025	N158K + K189R	<40	40	40	<40	80	640	40	1280	40
A/NL/00024/2025	N158K + K189R	<40	40	40	<40	40	320	40	1280	40
A/NL/02175/2024	N158K + K189R	<40	40	40	<40	80	640	40	1280	40
A/NL/02182/2024	N158K + K189R	<40	40	40	<40	80	640	40	640	40
A/NL/02093/2024	N158K + K189R	<40	<40	<40	<40	40	320	<40	640	<40

CA, Catalonia; CR, Croatia; DC, District of Columbia; MA, Massachusetts; LI, Lisboa; NL, Netherlands; SL, Slovenia; SW, Switzerland; TH, Thailand. A/CA/NSVH102423723/2024 is abbrebviated as A/CA/NSVH/2024.

The antisera (columns) are designated according to the virus (rows) used to prepare the antiserum. An asterisk indicates that the virus – antiserum combination was not tested. When the highest concentration of antiserum did not inhibit hemagglutination, the titer is indicated as lower than the lowest dilution tested (e.g. <10). Homologous titers are shown in bold.
